# Assessment of Depression, Anxiety, Stress, Quality of Life, and Fatigue in Patients After a Cerebrovascular Accident

**DOI:** 10.7759/cureus.72051

**Published:** 2024-10-21

**Authors:** Poulomi A Ghosh, Suprakash Chaudhury, Shalesh Rohatgi

**Affiliations:** 1 Psychiatry, Dr. D. Y. Patil Medical College, Hospital and Research Centre, Dr. D. Y. Patil Vidyapeeth (Deemed-to-be University), Pune, IND; 2 Neurology, Dr. D. Y. Patil Medical College, Hospital and Research Centre, Dr. D. Y. Patil Vidyapeeth (Deemed-to-be University), Pune, IND

**Keywords:** alcohol, comorbidities, disability, neuropsychiatric problems, stroke

## Abstract

Background

A cerebrovascular accident, or stroke, is a neurological disorder. Those who suffer from a stroke not only face physical disabilities but also a range of psychological issues resulting from concerns about their current situation. The purpose of this study was to assess depression, anxiety, stress, and fatigue after a stroke, along with its effects on quality of life (QoL), degree of disability, and prevalence of various risk factors of stroke.

Materials and methods

Adult male and female patients who had experienced a stroke and came to the psychiatry or neurology outpatient departments were evaluated. A detailed stroke history was taken for them. The Stroke-Specific Quality of Life Scale (SSQOL) Scale, Depression Anxiety Stress Scale 21, Alcohol Use Disorders Identification Test C (AUDIT C) scale, Suicide Behaviors Questionnaire-Revised (SBQ-R) scale, Fatigue Severity Scale (FSS), and Modified Rankin Scale (mRS) were applied to assess the QoL, depression, anxiety, stress, alcohol use, suicidal behavior, fatigue, and the degree of disability, if any, after stroke.

Results

The study involved 100 stroke patients aged 18-60 years, with 82 males and 18 females. The mean age was 45.76 ± 8.54 years. 88% experienced an ischemic stroke, and 12% a hemorrhagic stroke. 68% had comorbidities, and alcohol use was present in 48% of patients. The QoL was lowest in the domains of social roles, upper extremity, family roles, and productivity. The study revealed that 67% of patients experienced depression, 63% anxiety, and 62% stress, with 40% having a high risk of suicidal behavior, 57% experiencing fatigue, and 67% disability. There was a significant positive correlation between lower QoL scores, higher depression, anxiety, stress, disability, suicidal ideation, fatigue, and factors such as increased age, female gender, recurrent stroke, psychiatric illness history, multiple comorbidities, and substance use.

Conclusion

Neuropsychiatric problems following a stroke are common and have serious consequences that significantly influence stroke survivors' QoL and ability to recover.

## Introduction

Stroke is the leading cause of adult disability globally, causing 144-258 deaths per 100,000 people annually. Stroke survivors face health issues like speech impairment, cognitive difficulties, and weakness, along with psychological problems such as anxiety about dying, powerlessness, and concerns over social roles due to worries about their current situation [1]. Patients' quality of life (QoL) is impacted by stroke due to the physical and psychological consequences that accompany it, including reduced mobility and activity levels, anxiety, stress, and despair [2]. Stroke patients with minimal physical disability may also experience reduced QoL due to their psychological state, which is significantly impacted by neuropsychiatric problems like depression, anxiety, and stress, common post-stroke symptoms [1]. Stroke patients with psychological and emotional deficits struggle with recovery, with a high prevalence of neuropsychiatric disorders potentially negatively impacting their post-stroke QoL. Fatigue, a severe symptom of stroke, is considered one of the worst symptoms by up to 72% of stroke survivors [3]. Though stroke is one of the most prevalent causes of disability, and neuropsychiatric disorders following stroke are quite common, there is a paucity of studies addressing the co-occurrence of various mental health issues for stroke patients in India. Very few studies have assessed multiple risk factors and various after-effects of stroke affecting the QoL simultaneously in one study. In view of the foregoing, this study was undertaken to assess the frequency and intensity of stress, anxiety, and depression symptoms in stroke patients and their effects on QoL, fatigue, degree of disability, and prevalence of various risk factors of stroke.

## Materials and methods

This cross-sectional, analytical study was conducted between October 2022 and December 2023 in the psychiatry and neurology department of a tertiary care hospital and research center in Pune, India.

Ethics

The study was conducted in accordance with the ethical standards of the responsible committee on human experimentation (institutional or regional) and with the 1975 Helsinki Declaration as revised in 2000. Ethical clearance was obtained from the Institutional Ethics Sub-Committee (vide letter no.: I.E.S.C./313/2022 dated September 28, 2022).

Sample size calculation

The sample size for the study was calculated using Fisher’s formula: n = {z^2^p(1-p)}/d^2^, where 'n' represents the required sample size, 'z' is the z-value corresponding to a 95% confidence level (1.96), 'p' is the estimated prevalence (6.1% based on a previous study [4]), and 'd' is the margin of error set at 5% (0.05). Substituting these values into the formula yields 1.96x1.96x 0.061x0.939/(0.05x 0.05) = 88.01. Based on this calculation, a sample size of 100 was deemed appropriate for the study.

Sample

By purposive sampling, the study included patients who had experienced an ischemic or hemorrhagic stroke within the last five years, as diagnosed by a neurologist, reporting to either the Psychiatry or Neurology outpatient department, with their consent. All patients who had a transient ischemic attack or cerebrovascular disease with no acute cerebral symptoms and patients who were non-communicative were excluded from the study.

Data collection tools

Socio-demographic and Clinical Proforma

This self-made proforma included questions about the demographic characteristics of the subjects, their alcohol habits, and a detailed stroke history.

Alcohol Use Disorders Identification Test C (AUDIT C)

It is a reliable tool for identifying hazardous drinkers, with high reliability (Cronbach's alpha of 0.98, correlation coefficient of 0.95, and ICC of 0.95) [5].

Stroke-Specific Quality of Life Scale (SSQoL)

This outcome measure evaluates the health-related QoL for stroke patients across 12 domains. The 49-item scale demonstrated strong internal reliability (Cronbach's α values ≥0.73), with moderate correlations between domains of known outcome measures (r^2^ range, 0.3 to 0.5) [6].

Depression Anxiety Stress Scale 21 (DASS 21)

The DASS scales are used to rate individuals' perceptions of situations over the past week, resulting in stress, anxiety, and depression scores. The scale demonstrated internal consistency (Cronbach's α: 0.761 to 0.906) and moderate correlations with ADHP-V mental health-related domains (-0.47 to -0.66), supporting convergent validity [7].

Modified Rankin Scale (mRS)

It is a widely used tool to evaluate a person's level of dependency or disability post-stroke. The mRS has moderate inter-rater reliability (ⱪ 0.56 to 0.78) and strong test-retest reliability (ⱪ=0.81 to 0.95). There is a substantial association between the Barthel index scale trial endpoints and mRS (r=0.89, P<0.001) [8].

The Suicide Behaviors Questionnaire-Revised (SBQ-R)

This 4-item tool is used to screen for thoughts and behaviors related to suicide. The SBQ-R has adequate internal consistency, with a Cronbach's alpha of 0.82 and composite reliability of 0.86 [9].

Fatigue Severity Scale (FSS)

This self-reported 9-item questionnaire is used to assess the extent to which fatigue interferes with specific tasks. The FSS demonstrated excellent internal consistency in stroke patients (Cronbach's alpha: 0.928), with a 0.742 test-retest reliability coefficient and a positive correlation with the SF-36v scale (r = -0.531, p = 0.002) [10].

Method

All participants received an explanation of the study's aim. Written informed consent was obtained from every participant enrolled in the study. They were interviewed, and sociodemographic and clinical data were recorded. A detailed stroke history was taken from them. AUDIT C, SSQoL, DASS 21, mRS, SBQ-R scale, and FSS were administered to them.

Statistical analysis

These scores were compiled, and appropriate statistical analysis was conducted using descriptive and inferential statistics with IBM SPSS software for Windows, Version 29.0.2.0. Spearman’s coefficient (rs) was used to perform correlations among various variables. Multiple linear regression analysis was applied to ascertain the predictors.

## Results

The study involved 100 patients, 82 males and 18 females, with a mean age of 45.76 ± 8.54 years. The minimum age was 27 years, and the maximum was 60 years. Table 1 provides sociodemographic information, Table 2 provides a clinical history of stroke patients, and Table 3 displays the distribution of neurological impairment post-stroke. Eight patients had a seizure history, four had a hypercoagulable disorder, 14 had depression, three had schizophrenia, and one had an anxiety disorder in the past.

**Table 1:** Sociodemographic characteristics of stroke patients included in the study.

Characteristics	Subcategory	Cases %
Age distribution	18-30	4%
	31-45	40%
	45-60	56%
Marital Status	Married	75%
	Unmarried	12%
	Widowed	13%
Domicile	Rural	27%
	Semi-Urban	55%
	Urban	18%
Education	No formal education	9%
	Primary/Middle/ High school certificate	45%
	Diploma/Graduate/Profession	46%
Occupation	Unemployed	6%
	Elementary/ Plant/ Craft/ Skilled Agriculture/ Market Sales workers	41%
	Clerks/ Technicians/ Professionals/Senior officials	53%
Income	9232-68961	71%
	68967-184370	27%
	≥184,376	2%
Socioeconomic Status	Lower class	5%
	Upper lower	24%
	Lower middle	22%
	Upper middle	46%
	Upper	3%
Type of Family	Nuclear	73%
	Joint	27%
Dominant Hand	Right	84%
	Left	16%

**Table 2:** Clinical history of the stroke.

Category	Subcategory	Cases %
Type of stroke	Ischemic	88%
	Hemorrhagic	12%
Type of onset	Acute	60%
	Chronic	11%
	Recurrent	29%
Number of recurrences	None	71%
	One time	10%
	Two time	13%
	Three-time	6%
Localization of stroke	One hemisphere/ local	74%
	Two hemisphere/ locals	20%
	Multiple/ 3 locals	6%
Symptoms (Neurologic impairment)	One symptom	22%
	Two symptoms	35%
	> 2 symptoms	43%

**Table 3:** Unresolved effects of the stroke (neurological impairment).

Neurological impairment	Cases %
Motor Impairment	95%
Right hemiparesis	28%
Left hemiparesis	28%
Quadriparesis	9%
Right hemiplegia	11%
Left hemiplegia	4%
Quadriplegia	15%
Hemianopia/Quadrantanopia/Scotoma/Diplopia	10%
Communication problems due to dysarthria, dysphasia	37%
Swallowing disorders	5%
Spasticity	2%
Bladder dysfunction (incontinence)	42%
Sexual dysfunction	4%
Attention deficit	2%
Behavioral changes	9%
Gait abnormalities	14%

Among the patients, 42 had multiple comorbidities, including diabetes mellitus (57%), hypertension (43%), hyperlipidemia (15%), ischemic and coronary artery disease (5%), obesity (6%), bronchial asthma (5%), and thyroid disorder (2%). A total of 20% of the patients had a history of only one substance use, while 31% had multiple substance uses: 48 patients were found to be drinking alcohol, 26 were using tobacco products, and 12 were smoking cigarettes. Most patients consumed 1-2 standard drinks of country or Indian-made foreign liquor, with ten requiring alcohol to calm anxiety or shake off hangovers. Six had hematemesis, 15 had melena, and five had alcohol withdrawal seizures. AUDIT C scoring revealed 10% of patients falling into the category of increasing risk of alcohol dependence (score 5-7) and 90% in the low-risk category (score 0-4).

As assessed by the SSQoL, the overall mean QoL score was 2.95 points, which is less than the average of three points. The QoL of the patients after stroke was lowest in the domains of social roles, upper extremity, family roles, and productivity, followed by self-care, energy, mood, mobility, and personality (Table 4).

**Table 4:** Scores of the stroke patients on the Stroke Specific Quality of Life Scale (SSQoL).

Domains (normal score)	Mean	Median	SD	Minimum	Maximum
Energy (3-15)	2.73	3	1.41500	1	5
Family Roles (3-15)	2.54	2	1.45655	1	5
Language (5-25)	3.78	5	1.58639	1	5
Mobility (6-30)	2.82	2	1.45029	1	5
Mood (5-25)	2.74	3	1.47133	1	5
Personality (3-15)	2.96	3	1.46827	1	5
Self-Care (5-25)	2.73	3	1.33336	1	5
Social Roles (5-25)	2.25	2	1.24803	1	5
Thinking (3-15)	3.43	4	1.51447	1	5
Upper Extremity (5-25)	2.44	2	1.27362	1	5
Vision (3-15)	4.36	5	1.15824	1	5
Work/Productivity (3-15)	2.69	3	1.25664	1	5

After a stroke, 67% of patients experienced depression, 63% experienced anxiety, and 62% experienced stress, with mild to severe symptoms in all three categories as assessed by the DASS-21 (Table 5).

**Table 5:** Depression Anxiety Stress Scale-21 (DASS-21) scores.

Levels (range of scores)	Depression - Cases %	Anxiety - Cases %	Stress - Cases %
Normal (0-9)	33%	37%	38%
Mild (10-13)	6%	0%	6%
Moderate (14-20)	14%	9%	13%
Severe (21-27)	7%	6%	16%
Extremely Severe (28+)	40%	48%	27%

The mRS showed a mean score of 2.6 points, with 67% of the patients having some degree of disability ranging from slightly disabled to severe disability (Table 6).

**Table 6:** Modified Rankin Scale (mRS) scores.

Score	Interpretation	Cases %
0	No symptoms at all	1%
1	No significant disability despite symptoms	32%
2	Slight disability	17%
3	Moderate disability	17%
4	Moderately severe disability	22%
5	Severe disability	11%
6	Dead	0%

The SBQ-R revealed that 40% of patients had a score ≥ 7, indicating a significant risk of suicidal behavior. The mean (± SD) score of the stroke patients on the FSS was 37.19 ± 16.92, with 57% of patients having a fatigue score of at least 36, suggesting fatigue in the patients and the need for further evaluation by a physician. The mean scores of all the scales applied are detailed in Table 7.

**Table 7:** Scores obtained by stroke patients on the SSQoL, DASS-21, mRS, SBQ-R, FSS, and AUDIT C. SSQoL: Stroke Specific Quality of Life Scale; DASS-21: Depression Anxiety Stress Scale-21; mRS: Modified Rankin Scale; SBQ-R: Suicide Behaviors Questionnaire-Revised; FSS: Fatigue Severity Scale; AUDIT C: Alcohol Use Disorders Identification Test C.

Scales	Mean	Median	SD	Minimum	Maximum
Stroke Specific Quality of Life Scale	142.94	147.50	55.642	50	232
DASS 21 - Depression	20.66	20.00	15.371	0	42
DASS 21 - Anxiety	17.42	18.00	14.648	0	42
DASS 21 - Stress	21.24	23.00	14.072	0	42
Modified Rankin Scale	2.6	2.50	1.435	0	5
Suicide Behaviors Questionnaire-Revised	6.55	4.50	4.195	3	18
Fatigue Severity Scale	37.19	40.00	16.923	9	63
AUDIT C	1.420	0.00	1.934	0	7

QoL scores were positively correlated with higher socioeconomic status and negatively correlated with increasing age, female gender, unmarried status, past psychiatric illness, recurrent stroke, multiple localizations of stroke, comorbidities, and substance use, along with depression, anxiety, stress, scores of mRS, SBQ-R, FSS, and AUDIT-C scales.

A statistically significant positive correlation was observed between the scores for depression, anxiety, and stress on DASS 21 and factors such as age, female gender, unmarried status, recurrent stroke, past psychiatric illness, comorbidities, substance use, and scores of mRS, SBQ-R, FSS, and AUDIT-C. The scores of depression, anxiety, and stress were negatively correlated with QoL scores.

Suicidal ideation scores showed a positive correlation with female gender and depression. Disability scores were positively correlated with age and negatively correlated with QoL scores. FSS scores were positively correlated with age and depression. All Spearman correlation coefficients (rs) and corresponding P values are mentioned in Table 8.

**Table 8:** Correlation between the SSQoL, DASS-21, mRS, SBQ-R, FSS, and sociodemographic variables, clinical stroke history, comorbidity, substance use, and all other psychosocial scales used (Spearman’s Rho and p-value). SSQoL: Stroke-Specific Quality of Life Scale; DASS-21: Depression Anxiety Stress scale-21; mRS: Modified Rankin Scale; SBQ-R: Suicide Behaviors Questionnaire-Revised; FSS: Fatigue Severity Scale; AUDIT C: Alcohol Use Disorders Identification Test C.

Variables	SSQoL	DASS-21 Depression	DASS-21 Anxiety	DASS-21 Stress	mRS	SBQ-R	FSS
Age	r_s_ -0.641, P<0.0001	r_s_ 0.593, P<0.0001	r_s_ 0.428, P<0.0001	r_s_ 0.544, P<0.0001	r_s_ 0.677, P<0.0001	r_s_ 0.631, P<0.0001	r_s_ 0.754, P<0.0001
Sex	r_s_ -0.274, P<0.006	r_s_ 0.346, P<0.0001	r_s_ 0.431, P<0.0001	r_s_ 0.422, P<0.0001	r_s_ 0.158, P<0.117	r_s_ 0.341, P<0.001	r_s_ 0.260, P<0.009
Marital status	r_s_ -0.293, P<0.003	r_s_ 0.298, P<0.003	r_s_ 0.360, P<0.0001	r_s_ 0.316, P<0.001	r_s_ 0.276, P<0.005	r_s_ 0.339, P<0.001	r_s_ 0.246, P<0.014
Socio-economic status	r_s_ 0.517, P<0.0001	r_s_ -0.583, P<0.0001	r_s_ -0.510, P<0.0001	r_s_ -0.567, P<0.0001	r_s_ -0.406, P<0.0001	r_s_ -0.523, P<0.0001	r_s_ -0.505, P<0.0001
Past history of psychiatric illness	r_s_ -0.507, P<0.0001	r_s_ 0.505, P<0.0001	r_s_ 0.444, P<0.0001	r_s_ 0.500, P<0.0001	r_s_ 0.449, P<0.0001	r_s_ 0.490, P<0.0001	r_s_ 0.452, P<0.0001
No. of recurrences	r_s_ -0.635, P<0.0001	r_s_ 0.624, P<0.0001	r_s_ 0.469, P<0.0001	r_s_ 0.566, P<0.0001	r_s_ 0.649, P<0.0001	r_s_ 0.617, P<0.0001	r_s_ 0.652, P<0.0001
Localization of stroke	r_s_ -0.645, P<0.0001	r_s_ 0.620, P<0.0001	r_s_ 0.532, P<0.0001	r_s_ 0.608, P<0.0001	r_s_ 0.662, P<0.0001	r_s_ 0.692, P<0.0001	r_s_ 0.543, P<0.0001
Comorbidity	r_s_ -0.622, P<0.0001	r_s_ 0.540, P<0.0001	r_s_ 0.381, P<0.0001	r_s_ 0.475, P<0.0001	r_s_ 0.688, P<0.0001	r_s_ 0.542, P<0.0001	r_s_ 0.678, P<0.0001
Substance use	r_s_ -0.400, P<0.0001	r_s_ 0.418, P<0.0001	r_s_ 0.359, P<0.0001	r_s_ 0.365, P<0.0001	r_s_ 0.347, P<0.0001	r_s_ 0.333, P<0.001	r_s_ 0.344, P<0.0001
SSQOL	r_s_ 1.000	r_s_ -0.928, P<0.0001	r_s_ -0.812, P<0.0001	r_s_ -0.886, P<0.0001	r_s_ -0.897, P<0.0001	r_s_ -0.861, P<0.0001	r_s_ -0.853, P<0.0001
DASS-21 Depression	r_s_ -0.928, P<0.0001	r_s_ 1.000	r_s_ 0.872, P<0.0001	r_s_ 0.943, P<0.0001	r_s_ 0.825, P<0.0001	r_s_ 0.877, P<0.0001	r_s_ 0.814, P<0.0001
DASS-21 Anxiety	r_s_ -0.812, P<0.0001	r_s_ 0.872, P<0.0001	r_s_ 1.000	r_s_ 0.896, P<0.0001	r_s_ 0.698, P<0.0001	r_s_ 0.770, P<0.0001	r_s_ 0.711, P<0.0001
DASS-21 Stress	r_s_ -0.886, P<0.0001	r_s_ 0.943, P<0.0001	r_s_ 0.896, P<0.0001	r_s_ 1.000	r_s_ 0.772, P<0.0001	r_s_ 0.851, P<0.0001	r_s_ 0.783, P<0.0001
mRS	r_s_ -0.897, P<0.0001	r_s_ 0.825, P<0.0001	r_s_ 0.698, P<0.0001	r_s_ 0.772, P<0.0001	r_s_ 1.000	r_s_ 0.803, P<0.0001	r_s_ 0.803, P<0.0001
SBQ-R	r_s_ -0.861, P<0.0001	r_s_ 0.877, P<0.0001	r_s_ 0.770, P<0.0001	r_s_ 0.851, P<0.0001	r_s_ 0.803, P<0.0001	r_s_ 1.000	r_s_ 0.772, P<0.0001
FSS	r_s_ -0.853, P<0.0001	r_s_ 0.814, P<0.0001	r_s_ 0.711, P<0.0001	r_s_ 0.783, P<0.0001	r_s_ 0.803, P<0.0001	r_s_ 0.772, P<0.0001	r_s_ 1.000
AUDIT C	r_s_ -0.354, P<0.0001	r_s_ 0.395, P<0.0001	r_s_ 0.352, P<0.0001	r_s_ 0.342, P<0.0001	r_s_ 0.348, P<0.0001	r_s_ 0.310, P<0.002	r_s_ 0.313, P<0.001

Ischemic stroke, worse QoL scores, impact across multiple brain areas, and having several comorbidities are major predictors of the degree of disability. Suicidal behavior was strongly predicted by advanced age, being unmarried or widowed, having a stroke that affected many brain regions, and having a higher depression score (Table 9).

**Table 9:** Multiple regression analysis for predictors of degree of disability (mRS scale score) and suicidal ideation (SBQ-R scale score): coefficients. SSQoL: Stroke-Specific Quality of Life Scale; Comorbidity: No. of comorbidities in patients; Localization: Localization of stroke; Depression scores: DASS-21; Marital status: Marital status of the patient; Age: The age of the patient.

Dependent variables		Predictors	Unstandardized Coefficients		Standardized Coefficients	t	Sig.	95.0% CI for B		Collinearity Statistics	
			B	Std. Error	Beta			Lower Bound	Upper Bound	Tolerance	VIF
Degree of disability	(Constant)		4.719	0.456	N/A	10.355	0.000	3.814	5.624	N/A	N/A
	SSQoL		-0.019	0.002	-0.719	-12.210	0.000	-0.022	-0.016	0.450	2.222
	Comorbidity		0.243	0.086	0.145	2.818	0.006	0.072	0.413	0.589	1.698
	Type of stroke		-0.479	0.177	-0.109	-2.714	0.008	-0.830	-0.129	0.967	1.035
	Localization		0.337	0.128	0.137	2.634	0.010	0.083	0.591	0.576	1.735
Suicidal ideations	(Constant)		-3.37	1.035	N/A	-3.257	0.002	-5.427	-1.316	N/A	N/A
	Localization		1.960	0.389	0.273	5.034	0.000	1.187	2.732	0.552	1.810
	Depression scores		0.151	0.015	0.554	9.818	0.000	0.121	0.182	0.510	1.963
	Marital status		0.898	0.269	0.151	3.334	0.001	0.363	1.433	0.787	1.271
	Age		0.065	0.027	0.132	2.398	0.018	0.011	0.119	0.534	1.874

## Discussion

In the present study of 100 post-stroke patients, we found that 82% were men and 18% were women, a finding consistent with previous research, which reported a 25% higher risk of stroke in men [11]. The observation that the majority of stroke patients were aged 45-60 years aligns with other research revealing that a third of stroke patients are aged 45-65 [12].

Ischemic stroke was observed in 88% of stroke patients, with acute onset in 60%, indicating ischemic infarctions as the most prevalent stroke subtype [13]. About one-third of stroke patients experienced a second stroke, in agreement with an earlier study [14]. In our study, stroke patients frequently experienced movement impairment (95%), urine incontinence (42%), and communication issues (37%), which aligns with previous findings showing upper limb weakness (77%) and bladder control issues (50%) as common deficits [15].

Comorbidities like hypertension, diabetes, cancer, and kidney diseases are prevalent in 75%-99% of stroke populations [16]. Our study found that 57% of patients had diabetes and 43% had hypertension. Diabetes is a key risk factor for stroke, contributing to 5% of stroke risk in the population. Hypertension significantly increases stroke risk, with 72% of stroke patients having a history of hypertension and up to four times higher risk than those with normal blood pressure. Stroke risk rises by 20% for every 10 mmHg increase in systolic blood pressure. Long-term high blood pressure damages blood vessels, narrows arteries, and increases ischemic stroke risk, especially when combined with diabetes [16].

Our study found that 15% of patients had high cholesterol, a known risk factor for atherosclerosis and stroke, consistent with other research showing that 25% to 40% of stroke victims have high cholesterol [17].

The study found that 10% of 48 patients with a history of alcohol use were at higher risk of dependence, with increased alcohol consumption positively linked to stroke risk. Drinking over two drinks daily and binge drinking are independent stroke risk factors, especially for ischemic stroke. Chronic alcohol use also weakens blood vessels, raising hemorrhagic stroke risk. Episodic heavy drinking can cause arrhythmias and blood pressure spikes, leading to stroke from heart thrombi and emboli formation [18].

Stroke significantly impacts patients' quality of life (QoL), health, and well-being, affecting their perception of independence, responsibility, and meaningful relationships. Assessing QoL after a stroke considers physical, functional, mental, and social domains. A summary score of 143 in our study indicates that most stroke patients are dissatisfied with their QoL post-stroke, consistent with previous studies [2]. The domains with the lowest QoL scores were those related to social and familial duties, upper extremity functions, productivity, self-care, energy, and mood. Low mobility and upper extremity difficulties from neurological impairments hinder daily activities like transportation, self-care, and competency. Rehabilitation improves motor function and quality of life in post-stroke patients [2].

QoL after stroke is influenced by factors like social background, impairment, disability, mood, and support. Elderly stroke patients face worse morbidity, mortality, and long-term outcomes, leading to significantly poorer QoL compared to younger individuals [12]. Younger people strive to improve their status, while older individuals focus less on important tasks. The study suggests reducing stroke prevalence early, focusing on lifestyle modification, modifiable risk factors, and a balanced lifestyle to improve quality of life. It also highlights that QoL after a stroke is influenced by gender, with men showing better outcomes and functional performance [2]. The study emphasizes the importance of social support and marital status in patients' QoL. Social support enhances psychological well-being, motivation, and mental stability in long-term disability patients. It provides financial, physical, and emotional support, enhances self-care, promotes overall well-being, and inspires hope. Stroke recurrence negatively impacts patient morbidity and mortality, leading to lower QoL [2].

The study reveals that neuropsychiatric problems following a stroke are prevalent and can significantly impact rehabilitation outcomes, disability, and even death. The most common post-stroke mental health symptoms are depression (67%), anxiety (63%), and stress (62%) in our study. Depression affects 22% to 40% of stroke victims globally, peaking within six months and ranging from 20% to 50% in the first year [19]. Anxiety and stress disorders are prevalent in 9.4% to 36.7% and 31%, respectively [20,21].

Half of stroke survivors experience significant depression in the first year, often feeling hopeless and uncertain about their future [19]. Depression after stroke, characterized by high death rates, recurrence, and poor cognitive outcomes, is caused by factors like lesion site, neurotransmitter anomalies, inflammation, and social disability. Biological mechanisms involve brain damage, while psychological mechanisms involve inadequate coping mechanisms. Stroke poses a danger for suicide and suicidal thoughts as well. In our study, there was a considerable risk of suicidal behavior in 40% of the patients. Stroke increases suicide risk in women and young adults, with post-stroke depression, past mental disorders, stroke history, and cognitive impairment being the risk factors for suicide among survivors [22]. Stroke patients also experience high anxiety, ranging from 20% within one month to 24% after six months [23]. Additionally, 75% of stroke patients express significant anxiety about another stroke [23]. Our study found that stroke survivors often experience high-stress levels, which are linked to poorer long-term outcomes. Stress can cause functional independence loss and psychological effects like depression and cognitive decline, leading to long-term disability and motor and cognitive impairment [21].

The study revealed that individuals with higher stress, anxiety, and depression scores are more likely to be older, female, live in rural areas, have lower income, have multiple brain locations affected by recurrent stroke, have past psychiatric illnesses and comorbidities, and use illicit substances. Lesion location can indicate depression severity, with anterior left hemisphere injuries being more common due to a higher correlation with catecholamine depletion. Research indicates that female gender significantly predicts depression after stroke, possibly due to differences in brain architecture, performance, and biological and hormonal factors between the sexes [24]. Another study supports the importance of maintaining interpersonal relationships and social engagement in individuals with chronic illnesses [25]. Neuropsychiatric issues and depression significantly worsen stroke survivors' quality of life and increase mortality and recurrence beyond stroke severity.

Stroke leaves one-third of survivors fully recovered, another third dependent, and the rest with ongoing impairments. Dependency on daily activities is highest right after a stroke but gradually decreases. Our study revealed that 67% of patients had some form of disability, with 11% experiencing severe disabilities that required constant nursing and care. Research indicates that while 50-70% of stroke survivors recover independently, 15-20% remain permanently handicapped, and 20% require hospital care post-stroke. 87% of long-term survivors also have ongoing motor impairments [26]. Our study shows that patients with greater disability scores have worse QoL. Studies indicate a significant link between functional and physical capacity and daily living activities, impacting patients' QoL. Stroke disability is influenced by patient-level factors like age, comorbidities, and stroke severity.

Fatigue, a common post-stroke symptom, is defined as chronic and excessive exhaustion, affecting 39% to 72% of patients [3]. Our study found post-stroke fatigue in 57% of patients. Research shows that 68% of stroke survivors experience fatigue from neurological and psychosocial issues, sleep problems, and cognitive effects. In a 75-year-old population, 72% experienced mental fatigability, indicating astheno-emotional syndrome [27].

Post-stroke fatigue significantly impacts disability rates and health-related quality of life (HRQoL), potentially worsened by depression symptoms and motor impairment. Studies show a link between depression and fatigue, with 38% of fatigued patients experiencing high levels of depression [28].

Thus, post-stroke patients often face overlooked mental health issues, which require effective therapy, including selective serotonin reuptake inhibitors (SSRIs), serotonin and norepinephrine reuptake inhibitors (SNRIs), and cognitive behavioral therapy (CBT), to prevent neuropsychiatric problems and ensure proper treatment [29]. Stroke rehabilitation is crucial for reintegration into society, focusing on psychosocial adaptation and functional independence to increase life satisfaction and QoL [30].

Strengths

Stroke is a prevalent cause of disability and neuropsychiatric issues, yet there is a lack of research on the co-occurrence of mental health issues in stroke patients in India and other developing nations. This study analyzed various factors, including quality of life, depression, anxiety, stress, suicidal behavior, disability, fatigue, and stroke risk factors simultaneously, aiming to identify significant predictors of neuropsychiatric illnesses and the correlation between risk factors and stroke residual effects, making it one of a kind.

Limitations

The sample of this study excluded individuals who were unable to communicate as a result of stroke sequelae; hence, the study cannot be generalized. A small number of patients reported pre-stroke psychiatric issues, which may have impacted the post-stroke mental health symptoms.

## Conclusions

Stroke survivors often face neuropsychiatric problems that affect their QoL and are frequently overlooked. Preventing and treating these illnesses can improve their prognosis, reduce social impairments, increase survival rates, and lower death rates during rehabilitation. Stroke prevention requires a multimodal approach, addressing both modifiable and non-modifiable risk factors, and emphasizing the dangers of excessive drinking. Lifestyle modifications, medication adherence, and routine monitoring can manage these risk factors, thereby reducing stroke incidence and improving public health outcomes. Education and awareness campaigns are crucial for reducing stigma and promoting help for neuropsychiatric illnesses.

## Appendices

Appendix 1

*Socio-Demographic Proforma, Stroke History Proforma, and Alcohol History Proforma*                                                                                    

1. Name:       

2. Age:

3. Sex:  

4. Marital status:

5. Residence/Domicile:

6. Educational Qualification:

7. Occupational status:

8. Monthly income of the family:

9. Socio economic status:

10. Type of family: 

11. Type of hand dominance:

12. Medical diagnosis of stroke:

13. Type of occurrence/onset of stroke - Acute/Chronic/Recurrent:

14. No. of recurrences, if any:

15. No. of Locations affected by stroke:

16. Specific locations affected by stroke:

17. No. of symptoms/unresolved effects of stroke (Neurological impairment):

18. Specific symptoms/unresolved effects of stroke (Neurological impairment):

19. No. of Co-morbidities present:

20. Specific Co-morbidities present:

21. Past history of psychiatric illness:

22. History of seizure, if any:

23. History of oral contraceptive use:

24. History of anticoagulant therapy:

25. History of any hypercoagulable disorder:

26. History of any antiepileptic drug use:

27. No. of Substances used:

28. Specific substances used:

29. History of consumption of alcohol:

30. The age at which alcohol consumption was started:

31. Type of alcohol consumed:

32. Daily consumption of how many standard drinks:

33. Time since last alcoholic drink consumed:

34. Need for a drink first thing in the morning (Eye-opener):

35. History of Hematemesis due to alcohol consumption:

36. History of Melena due to alcohol consumption:

37. History of Alcohol withdrawal seizure:

APPENDIX 2

Stroke-Specific Quality of Life Scale (SSQOL)

Energy

1. I felt tired most of the time. ____

2. I had to stop and rest during the day. ____

3. I was too tired to do what I wanted to do. ____

Family Roles

1. I didn't join in activities just for fun with my family. ____

2. I felt I was a burden to my family. ____

3. My physical condition interfered with my personal life. ____

Language

1. Did you have trouble speaking? For example, get stuck, stutter, stammer, or slur your words? ____

2. Did you have trouble speaking clearly enough to use the telephone? ____

3. Did other people have trouble in understanding what you said? ____

4. Did you have trouble finding the word you wanted to say? ____

5. Did you have to repeat yourself so others could understand you? ____

Mobility

1. Did you have trouble walking? (If patient can't walk, go to question 4 and score questions 2-3 as 1.) ____

2. Did you lose your balance when bending over to or reaching for something? ____

3. Did you have trouble climbing stairs? ____

4. Did you have to stop and rest more than you would like when walking or using a wheelchair? ____

5. Did you have trouble with standing? ____

6. Did you have trouble getting out of a chair? ____

Mood

1. I was discouraged about my future. ____

2. I wasn't interested in other people or activities. ____

3. I felt withdrawn from other people. ____

4. I had little confidence in myself. ____

5. I was not interested in food. ____

Personality

1. I was irritable. ____

2. I was inpatient with others. ____

3. My personality has changed. ____

Self-Care

1. Did you need help preparing food? ____

2. Did you need help eating? For example, cutting food or preparing food? ____

3. Did you need help getting dressed? For example, putting on socks or shoes, buttoning buttons, or zipping? ____

4. Did you need help taking a bath or a shower? ____

5. Did you need help to use the toilet? ____

Social Roles

1. I didn't go out as often as I would like. ____

2. I did my hobbies and recreation for shorter periods of time than I would like. ____

3. I didn't see as many of my friends as I would like. ____

4. I had sex less often than I would like. ____

5. My physical condition interfered with my social life. ____

Thinking

1. It was hard for me to concentrate. ____

2. I had trouble remembering things. ____

3. I had to write things down to remember them. ____

Upper Extremity Function

1. Did you have trouble writing or typing? ____

2. Did you have trouble putting on socks? ____

3. Did you have trouble buttoning buttons? ____

4. Did you have trouble zipping a zipper? ____

5. Did you have trouble opening a jar? ____

Vision

1. Did you have trouble seeing the television well enough to enjoy a show? ____

2. Did you have trouble reaching things because of poor eyesight? ____

3. Did you have trouble seeing things off to one side? ____

Work/Productivity

1. Did you have trouble doing daily work around the house? ____

2. Did you have trouble finishing jobs that you started? ____

3. Did you have trouble doing the work you used to do? ____

TOTAL SCORE ____

Scoring: Items are rated on a 5-point Likert scale. 

1 - Total help/Couldn’t do it at all/Strongly agree

2 - A lot of help/A lot of trouble/Moderately agree

3 - Some help/Some trouble/Neither agree nor disagree

4 - A little help/A little trouble/Moderately disagree

5 - No help needed/No trouble at all/Strongly disagree

Higher scores indicate better functioning. The SS-QOL yields both domain scores and an overall SS-QOL summary score. The domain scores are unweighted averages of the associated items, while the summary score is an unweighted average of all twelve domain scores.

Appendix 3

Depression Anxiety Stress Scale 21 (DASS-21)

The rating scale is as follows:

 0 - Did not apply to me at all

 1 - Applied to me to some degree, or some of the time

 2 - Applied to me to a considerable degree or a good part of time

 3 - Applied to me very much or most of the time

1.(s) I found it hard to wind down 2.(a) I was aware of dryness of my mouth 3.(d) I couldn’t seem to experience any positive feeling at all 4.(a) I experienced breathing difficulty (e.g. excessively rapid breathing, breathlessness in the absence of physical exertion) 5.(d)I found it difficult to work up the initiative to do things 6.(s) I tended to over-react to situations 7.(a) I experienced trembling (e.g. in the hands) 8.(s)I felt that I was using a lot of nervous energy 9.(a) I was worried about situations in which I might panic and make a fool of myself 10.(d) I felt that I had nothing to look forward to 11.(s) I found myself getting agitated 12.(s) I found it difficult to relax 13.(d) I felt down-hearted and blue 14.(s) I was intolerant of anything that kept me from getting on with what I was doing 15.(a) I felt I was close to panic 16.(d) I was unable to become enthusiastic about anything 17.(d) I felt I wasn’t worth much as a person 18.(s) I felt that I was rather touchy 19.(a) I was aware of the action of my heart in the absence of physical exertion (e.g. sense of heart rate increase, heart missing a beat) 20.(a) I felt scared without any good reason 21.(d) I felt that life was meaningless

DASS-21 Scoring Instructions:

The Depression, Anxiety, and Stress Scale - 21 Items (DASS-21) is a set of three self-report scales designed to measure the emotional states of depression, anxiety, and stress. Each of the three DASS-21 scales contains 7 items, divided into subscales with similar content. The depression scale assesses dysphoria, hopelessness, devaluation of life, self-deprecation, lack of interest/involvement, anhedonia, and inertia. The anxiety scale assesses autonomic arousal, skeletal muscle effects, situational anxiety, and subjective experience of anxious affect. The stress scale is sensitive to levels of chronic nonspecific arousal. It assesses difficulty relaxing, nervous arousal, being easily upset/agitated, irritable /over-reactive, and impatient. Scores for depression, anxiety, and stress are calculated by summing the scores for the relevant items. Recommended cut-off scores for conventional severity labels (normal, moderate, severe) are as follows:

1) Depression Score

a. Normal: 0-9

b. Mild: 10-13

c. Moderate: 14-20

d. Severe: 21-27

e. Extremely Severe: 28+

2) Anxiety Score

a. Normal: 0-7

b. Mild: 8-9

c. Moderate: 10-14

d. Severe: 15-19

e. Extremely Severe: 20+

3) Stress Score

a. Normal: 0-14

b. Mild: 15-18

c. Moderate: 19-25

d. Severe: 26-33

e. Extremely Severe: 34+

Scores on the DASS-21 will need to be multiplied by 2 to calculate the final score.

Appendix 4

The Suicide Behaviors Questionnaire-Revised (SBQ-R)

The SBQ-R has 4 items‚ each tapping a different dimension of suicidality:

Item 1 taps into lifetime suicide ideation and/or suicide attempt.

Item 2 assesses the frequency of suicidal ideation over the past twelve months.

Item 3 assesses the threat of Suicide attempt.

Item 4 evaluates self-reported likelihood of suicidal behavior in the future.

1)     Have you ever thought about or attempted to kill yourself? (Check one only)

1. Never

2. It was just a brief passing thought

3a. I have had a plan at least once to kill myself but did not try to do it

3b. I have had a plan at least once to kill myself and really wanted to die

4a. I have attempted to kill myself‚ but did not want to die

4b. I have attempted to kill myself‚ and really hoped to die

2)   How often have you thought about killing yourself in the past year? (Check one only)

1. Never

2. Rarely (1 time)

3. Sometimes (2 times)

4. Often (3-4 times)

5. Very Often (5 or more times)

3)     Have you ever told someone that you were going to commit suicide‚ or that you might do it? (Check one only)

1. No

2a. Yes‚ at one time‚ but did not really want to die

2b. Yes‚ at one time‚ and really wanted to die

3a. Yes‚ more than once‚ but did not want to do it

3b. Yes‚ more than once‚ and really wanted to do it

4)     How likely is it that you will attempt suicide someday? (Check one only)

0. Never

1. No chance at all

2. Rather unlikely

3. Unlikely

4. Likely

5. Rather likely

6. Very likely

Scoring: The SBQ-R is scored on a scale of 3 to 18, with higher scores indicating a higher risk of suicidal behaviors. According to previous studies, the cut-off value for suicide risk was 7 in the general adult population.

Appendix 5

Modified Rankin Scale (mRS)

Score Description

0 - No symptoms at all

1 - No significant disability despite symptoms; able to carry out all usual duties and activities

2 - Slight disability; unable to carry out all previous activities, but able to look after own affairs without assistance

3 - Moderate disability; requiring some help, but able to walk without assistance

4 - Moderately severe disability; unable to walk without assistance and unable to attend to own bodily needs without assistance

5 - Severe disability; bedridden, incontinent and requiring constant nursing care and attention

6 - Dead 

 TOTAL (0-6): _______

The Modified Rankin Score (mRS) is a 6-point disability scale with possible scores ranging from 0 to 5. A separate category of 6 is usually added for dead patients.

Appendix 6

Fatigue Severity Scale (FSS)

1. My motivation is lower when I am fatigued.

2. Exercise brings on my fatigue.

3. I am easily fatigued.

4. Fatigue interferes with my physical functioning.

5. Fatigue causes frequent problems for me.

6. My fatigue prevents sustained physical functioning.

7. Fatigue interferes with carrying out certain duties and responsibilities.

8. Fatigue is among my most disabling symptoms.

9. Fatigue interferes with my work, family, or social life.

Scoring: The items are scored on a 7-point scale with

1 = strongly disagree and 7= strongly agree.

The higher the score, the greater the fatigue severity.

A total score of 36 or more suggests fatigue and suggests that one may need further evaluation by a physician.

Appendix 7

Alcohol Use Disorders Identification Test C (AUDIT C)

1. Within the past year, how often did you have a drink of alcohol? a. Never b. Monthly (e.g., Special occasions/Rare) c. 2-4 times a month (e.g., 1x on weekend - “Fridays only” or “every other Thursday”) d. 2-3 times a week (e.g., weekends - Friday-Saturday or Saturday-Sunday) e. 4 or more times a week (e.g., daily or most days/week)

2. Within the past year, how many standard drinks containing alcohol did you have on a typical day? a. 1 or 2 b. 3 or 4 c. 5 or 6 d. 7 to 9 e. 10 or more

3. Within the past year, how often did you have six or more drinks on one occasion? a. Never b. Less than monthly c. Monthly d. Weekly e. Daily or almost daily

Scoring: The AUDIT-C has 3 questions and is scored on a scale of 0-12. Each AUDIT-C question has 5 answer choices valued from 0 points to 4 points. In men, a score of 4 or more is considered positive, optimal for identifying hazardous drinking or active alcohol use disorders.
